# Prosociality moderates outcome evaluation in competition tasks

**DOI:** 10.1038/s41598-022-15570-3

**Published:** 2022-07-06

**Authors:** Jiachen Lu, Weidong Li, Yujia Xie, Qian Huang, Jingjing Li

**Affiliations:** 1 School of Psychology, South China Normal University, Guangzhou, 510631 China; 2 School of Physical Education, Guangzhou University, Guangzhou, 510006 China

**Keywords:** Motivation, Social behaviour, Social neuroscience

## Abstract

The current study investigated the effect of prosociality on outcome evaluation without involving social comparison and reward processing in face-to-face competition tasks. The results showed that when faced with medium and large outcome feedback, the feedback-related negativity (FRN) amplitude induced in high-prosocial individuals was significantly more negative than that of low-prosocial individuals. In addition, the P300 amplitude induced in high-prosocial individuals was smaller than that in low-prosocial individuals in the face of large outcome feedback; hence, the prosociality score was significantly correlated with FRN amplitude. However, there was no significant difference in FRN between high-and low-prosocial individuals in the face of small outcome feedback. It was concluded that individual prosocial traits can moderate outcome evaluation.

## Introduction

Outcome evaluation refers to the process in which the cognitive system makes rapid evaluations of the results caused by a person’s own behavior^1^. People can obtain relevant information about the appropriateness of their own responses through external feedback stimuli. The process of outcome evaluation is not only affected by the valence and size of the outcome^2^; simultaneously, it is affected by a variety of factors. These include peer participation^3–5^, action^6^, past experiences^7^, and personality traits^8,9^.

Prosociality, as an individual trait, is also a popular research topic in outcome evaluation^10^. Prosociality refers to the tendency to benefit others at the expense of oneself^11^. Two aspects are at the core of prosociality: working for the welfare of others and the absence of selfish motives. Self-interest is an unintended by-product, rather than the goal of prosociality^12^. Therefore, when the outcome of highly prosocial individuals involves the interests of others, the process of outcome evaluation not only involves the impact of the outcome on themselves but may also involve the impact of the outcome on others^13^.

A large amount of work using the event-related potential (ERP) technique has identified outcome evaluation-related components such as feedback-related negativity (FRN) and P300^8^. FRN is a negative-going deflection in the ERP occurring, which triggered at the frontal scalp and peaks in the prefrontal scalp approximately 250–300 ms after the feedback stimulus is initiated^14–17^. According to the reinforcement learning theory proposed by Holroyd and Coles^14^, the expected error signal (the actual result is different from the expected result) reduces the dopamine produced by the basal ganglia acting on anterior cingulate cortex (ACC) , and fails to inhibit the activity of ACC, resulting in a large FRN amplitude. When there is a positive expectation error (the actual outcome is better than expected), dopamine is increased, ACC activity is decreased and FRN amplitude is smaller. Whereas Holroyd and Coles^14^ interpreted the FRN purely as reinforcement signal, Gehring and Willoughby^18^ stated that the FRN might reflect the motivational impact of ongoing events. Previous studies have found that FRN components are related to prosociality^19–21^. For example, Gan et al.^10^ used ERP to explore the neural correlates of outcome evaluation for helping others during a decision-making task. Their results showed that the amplitude of FRN elicited by a failed outcome (indicating a failure in helping others) was larger than that elicited by a successful outcome (indicating success in helping others).

Preliminary evidence suggests that the P300 is also sensitive to prosociality and outcomes. The P300 is a positive ERP component that peaks at midline parietal sites about 200–600 ms after outcome feedback onset^22^. Its distribution is more widespread than that of FRN, and the anterior cingulate cortex and parietal cortex are considered to be key contributors to its neural generation (for review, see Linden^23^). The P300 appears to reflect the processes of attention allocation and the motivational/emotional salience of outcomes, such that more positive outcomes elicit a larger P300^6,24^. Ma et al.^25^ found a larger P300 amplitude when individuals looked at the monetary gains of others to whom they had emotional closeness (e.g., friends) compared to strangers. In addition, the P300 amplitude increased with the perceived needs of the target in the scenarios in which help is required, and situations involving targets with urgent, unattended needs (e.g., serious injury) triggered a larger P300 response than when no help was required^26^. The P300 activity was positively correlated with self-reported prosocial habits and implicit prosocial attitudes^27^.

Although there have been studies on the relationship between prosociality and outcome evaluation, their conclusions are not clear. In the past, most studies have focused on the evaluation of prosocial behavior outcomes or on the evaluation of other people’s reactions^25–27^. The relationship between individuals’ prosocial traits and outcome evaluation was not examined in previous research.

Moreover, psychological processes such as social comparison and rewards were usually involved in outcome evaluation^28–31^. Social comparison theory holds that humans have a tendency to compare their own abilities, wealth, and social status with others’ to gain knowledge about themselves^28^. Rewards involve a series of psychological processes, such as learning, emotions, and motivation^29^. Previous studies usually present outcome of comparison directly, involving the evaluation of others' outcome and reward^28,29^. It was found that both social comparison and reward significant affect FRN and P300 components^30,31^.Therefore, it is necessary to separate outcome of individual from that of comparison. In addition, in previous studies, participants usually completed tasks on their own or with a computer to control variables^32,33^. However, it is well known that the behavior and brain responses of participants differ when they interact with people and computers^34,35^.

Considering the above issues, this study used a face-to-face competition task. In this task, the outcomes of individual choice and the final reward were presented separately to separate the outcome evaluation from social comparison and reward. Individual prosocial traits were examined using questionnaires. These operations allowed the current study to explore the direct relationship between individual prosocial traits and outcome evaluation.

## Methods

### Participants

Forty participants (20 females and 20 males) with normal vision or corrected-to-normal vision and no psychological diseases participated in this study. The mean age of the participants was 22 years (SD = 2.13). Each task involved two participants of the same sex to eliminate any interference from gender^36^. This study was approved by the review committee of the South China Normal University, and the participants provided informed consent. All methods were performed in accordance with the relevant guidelines and regulations. Participants were compensated based on points earned during the task.

### Procedure

The experimental program was programmed using the E-prime software (version 3.0; Psychology Software Tools, Inc., Pittsburgh, PA, USA). All instructions and stimuli were displayed on a 23-inch screen with a gray background, resolution of 1280 × 768 pixels, and refresh rate of 60 Hz.

The two participants were seated in front of the monitor and were separated by a partition, they could both see the entire screen, but there was just a partition there so that they could not see the other person. They were required to complete a series of card selection tasks in order to compete for rewards. Participants simultaneously chose one of three cards on the screen, each concealing zero (*small* condition), one (*medium* condition), or two stars (*large* condition). The participant who selected the card with more stars gained 2 points, whereas the participant whose card had fewer stars lost 1 point. When they selected cards with the same number of stars, they were assigned no gain or loss.

**Figure 1:**
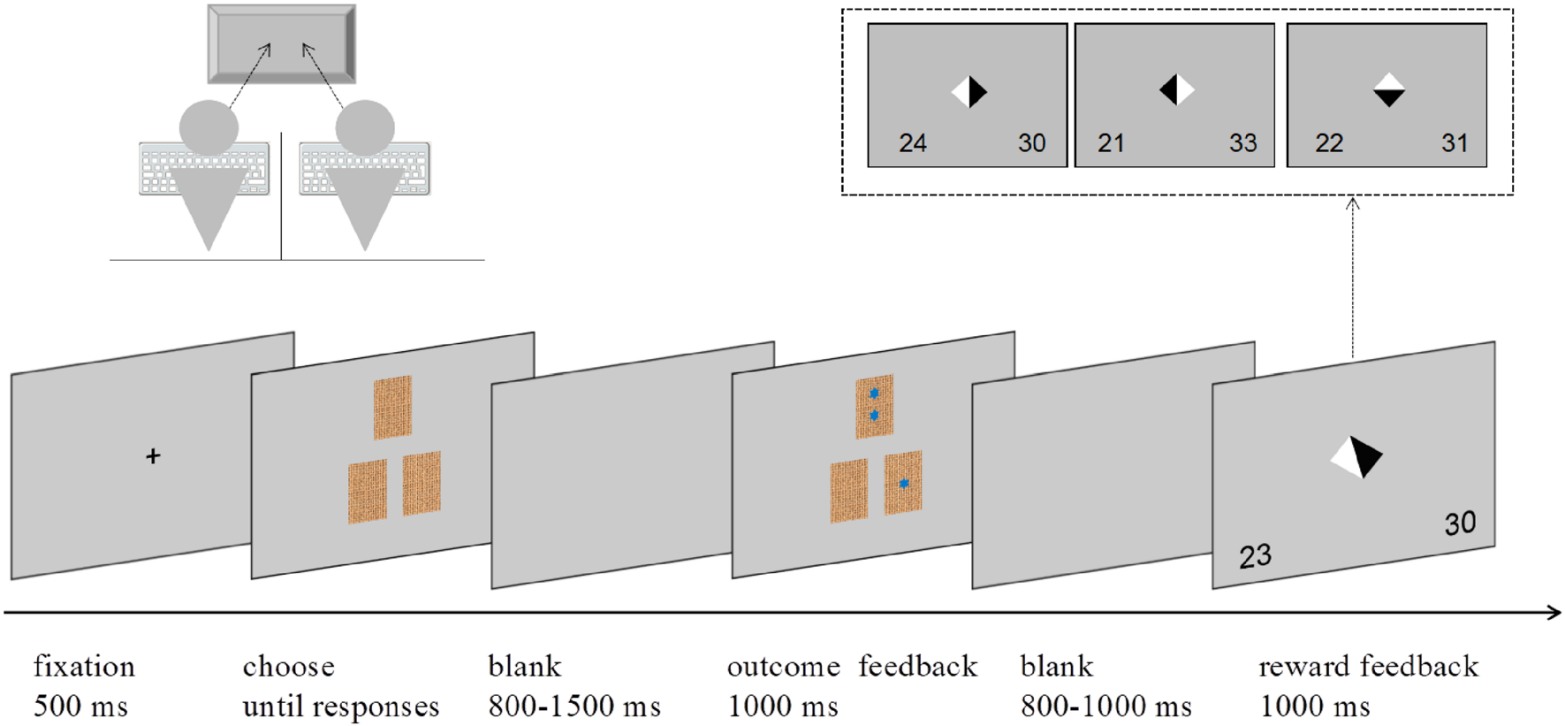
Experimental procedure. Two participants of the same sex compete in a reward task. The participants simultaneously chose one of three cards, concealing zero, one, or two stars. The person who selected the card with more stars gained a reward.

The process of a single trial is illustrated in Fig. 1. A fixation mark was presented for 500 ms, followed by the card selection array. In the selection array, three identical cards appeared on the upper, left, and right sides of the center of the screen. Participants were asked to choose one card using their respective keyboard. When both participants responded, following an inter-stimulus interval (ISI) of 800–1500 ms, the outcome feedback screen was presented for 1000 ms. The number of stars in each card was revealed on the outcome feedback screen. It's worth noting that when the outcome feedback was presented, the two participants did not know which card the other had selected. Following an inter-stimulus interval (ISI) of 800–1000 ms, the reward feedback screen was presented for 1000 ms. The reward feedback screen showed a black and white triangle, with the white triangle pointing to the participant who gained the reward (chose the card with more stars), and the black triangle pointing to the participant who lost the reward (chose the card with fewer stars). The black and white triangles were displayed pointing up and down rather than left/right to indicate that there was no gain or loss. Simultaneously, participants’ accumulated points were displayed in the lower left or right corner of the screen. The gap between the two adjacent tests was 1000 ms. Before the formal experiment, the participants performed 10 test exercises. Once participants understood the experimental process, the formal experiment began. The formal experiment consisted of four blocks of 72 trials each, with one to three minutes of rest between each block.

### The measure of prosocial scores

The Prosocial Scale^37^ was used to measure participants’ prosocial scores. This includes five items: *I usually share with others (games, food, pens, *etc*.); I try to be nice to other people; I care about their feelings; I am kind to younger children; I am helpful if someone is hurt, upset, or feeling ill; and I often volunteer to help others (parents, teachers, children)*. In order to prevent the influence between the questionnaire and the task^38^, the participants filled in the Prosocial Scale one week after finishing the experiment. For the Prosocial Scale, participants were asked to answer five items based on their experiences over the past six months, ranging from not true (1 point) to somewhat true (2 points) to certainly true (3 points). The higher the score, the higher the level of prosociality^39^.

### Electroencephalogram (EEG) recording and pretreatment

A cap with 64 electrodes (Brain Products, Munich, Germany) mounted according to the international 10/20 system was used to collect the EEG data . The forehead electrode, FCz, was used as the online reference point, and the AFz electrode was used as the ground electrode. A vertical electrooculogram (VEOG) was recorded using a facial electrode placed approximately 1.5 cm below the right eye. All electrodes were amplified using a 0.01–100 Hz online band-pass and continuously sampled at 1000 Hz/channel.

Offline EEG data were pre-processed using the EEGLAB toolbox^40^. First, re-referencing was performed for the bilateral mastoid TP9 and TP10, and all EEG signals were subjected to a 0.1-Hz high-pass wave, 30-Hz low-pass filtering, 50-Hz notch filtering, and vertical correction for blinks. Next, the period from 200 ms before to 1000 ms after the onset of the outcome feedback stimulus was analyzed for the FRN and P300 components, with the average amplitude from 0 to 200 ms before the outcome feedback stimulus onset as the corrected baseline. Any trial involving a peak-to-peak deflection exceeding ± 80 μV was excluded from the analysis.

### Data processing and statistical analysis

According to the prosocial scale score, the top 50% of the participants were divided into the high prosocial group (including 12 females), and the bottom 50% into the low prosocial group (including eight females). In order to prevent the outcome-induced ERP component from being caused by the actual probability of choices different types of cards, a 3 × 2 mixed design analysis of variance (ANOVA) was conducted. The true probability of the participants’ choices of card concealing different numbers of stars (small, medium, and large) as the within-subject variable and the group (high prosocial, low prosocial) as the between-subject variable.

For the ERP component, previous studies have reported that the FRN component mainly occurs in the front-middle forehead, and the peaks appear at 200–350 after the stimulus onset^3^, while the P300 component mainly occurs in the occipital region, and the peaks appear at 300–600 after the stimulus onset^33^. In addition, based on the waveforms in the current experiment, the mean amplitudes from 240 to 320 ms after the outcome feedback onset at the Fz, F1, F2, FCz, FC1, FC2, Cz, C1, and C2 electrodes were calculated for the FRN components, and the mean amplitudes from 360 to 460 ms after the outcome feedback presentation at the Cz, C1, C2, CPz, CP1, CP2, Pz, P1, and P2 electrodes were calculated for the P300 components, where the final value was taken as the average of the electrodes. Finally, a 3 size (small, medium, and large) × 2 group (high prosocial, low prosocial) repeated measures ANOVA was performed. In addition, Pearson’s correlation was used to calculate the correlation between the prosociality score and FRN amplitude to comprehensively reveal the relationship between prosociality and outcome evaluation. All simple effect analyses were performed using an independent sample t-test. Greenhouse–Geisser was used to correct the degrees of freedom for the ANOVA with uneven variance. And statistical significance was set at *p* < 0.05. All statistical analyses were conducted using the SPSS 22 software (IBM, USA).

### Informed consent

Informed consent was obtained from all subjects involved in the study.

## Results

### Behavioral results

**Figure 2:**
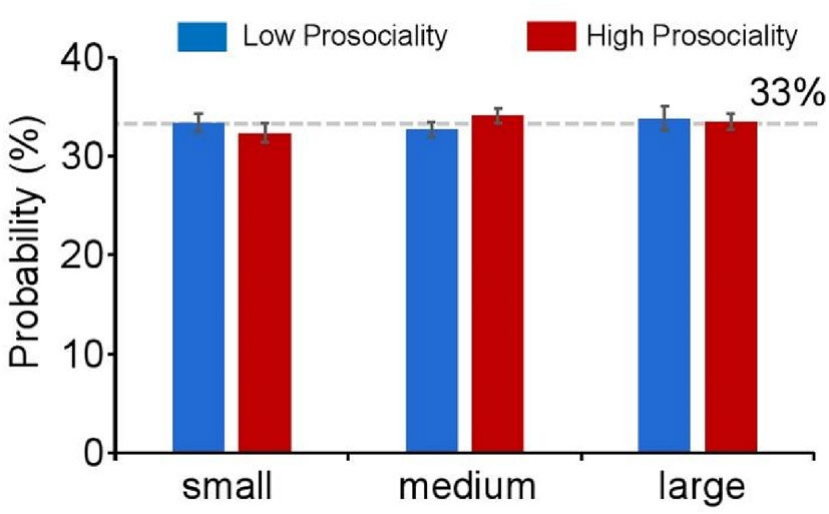
Behavioral results. The probabilities of each card choice are shown. Red is the high prosocial group and blue is the low prosocial group. Small: the individual choose a card containing 0 star. Medium: the individual choose a card containing 1 star. large: the individual choose a card containing 1 star.

No significant difference was found in the probability of *size* (F(2, 76) = 0.27, *p* = 0.77, *η*_*p*_^2^ = 0.01) (Fig. 2). No significant differences were found between *groups* (F(1, 38) = 0.40, *p* = 0.53, *η*_*p*_^2^ = 0.01). The interaction effect of *size* and *group* was not significant (F(2, 76) = 0.63, *p* = 0.53, *η*_*p*_^2^ = 0.02).

### ERP results

**Figure 3:**
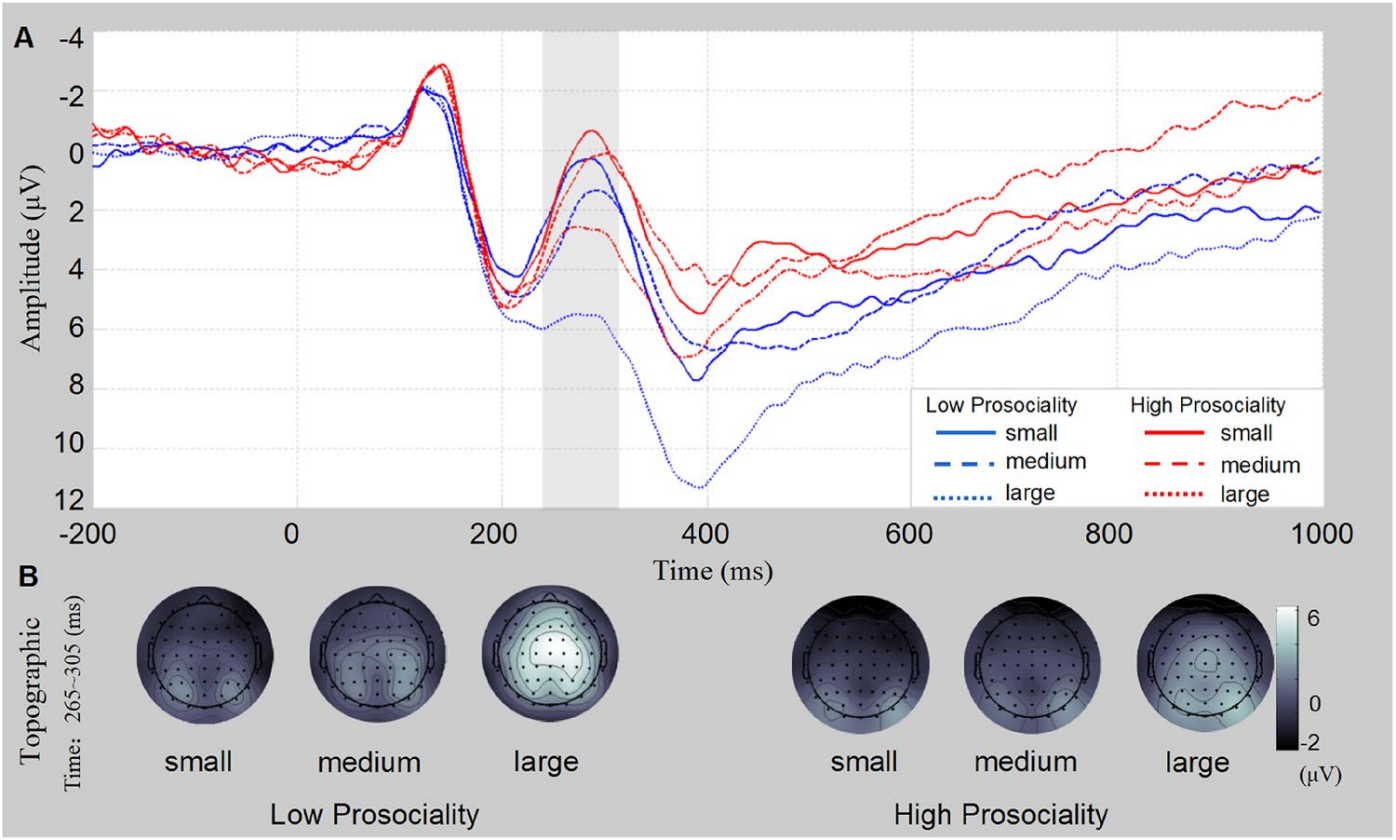
FRN results. (**A**) The mean waves of nine electrodes are shown. The gray area represents the selected time window. Small: the individual choose the card containing 0 star. Medium: the individual choose the card containing 1 star. Large: The individual choose the card containing 2 star. (**B**) The differential topographic map is shown.

For the FRN components (see Fig. 3), the main effect of *size* was significant (F(1.8, 52.4) = 41.07, *p* < 0.001, *η*_*p*_^2^ = 0.52), as was the main effect of *group* (F(1, 38) = 6.52, *p* = 0.02). *η*_*p*_^2^ = 0.15). In addition, the interaction between *group* and *size* was marginal significant (F(1.8, 52.4) = 3.40, *p* = 0.06, *η*_*p*_^2^ = 0.08). Simple effect analysis revealed that the amplitude of the high prosocial group under the large condition was significantly more negative that of the low prosocial group (*t*(38) = 3.20, *p* = 0.003, Cohen's *d* = 1.04), and the amplitude of the FRN in the high prosocial group under the medium condition was significantly more negative that of the low prosocial group (*t*(38) = 1.90, *p* = 0.03, Cohen’s *d* = 0.76). However, the difference between high and low prosocial groups was not significant under the small condition (*t*(38) = 1.47, *p* = 0.15, Cohen's *d* = 0.48).

**Figure 4:**
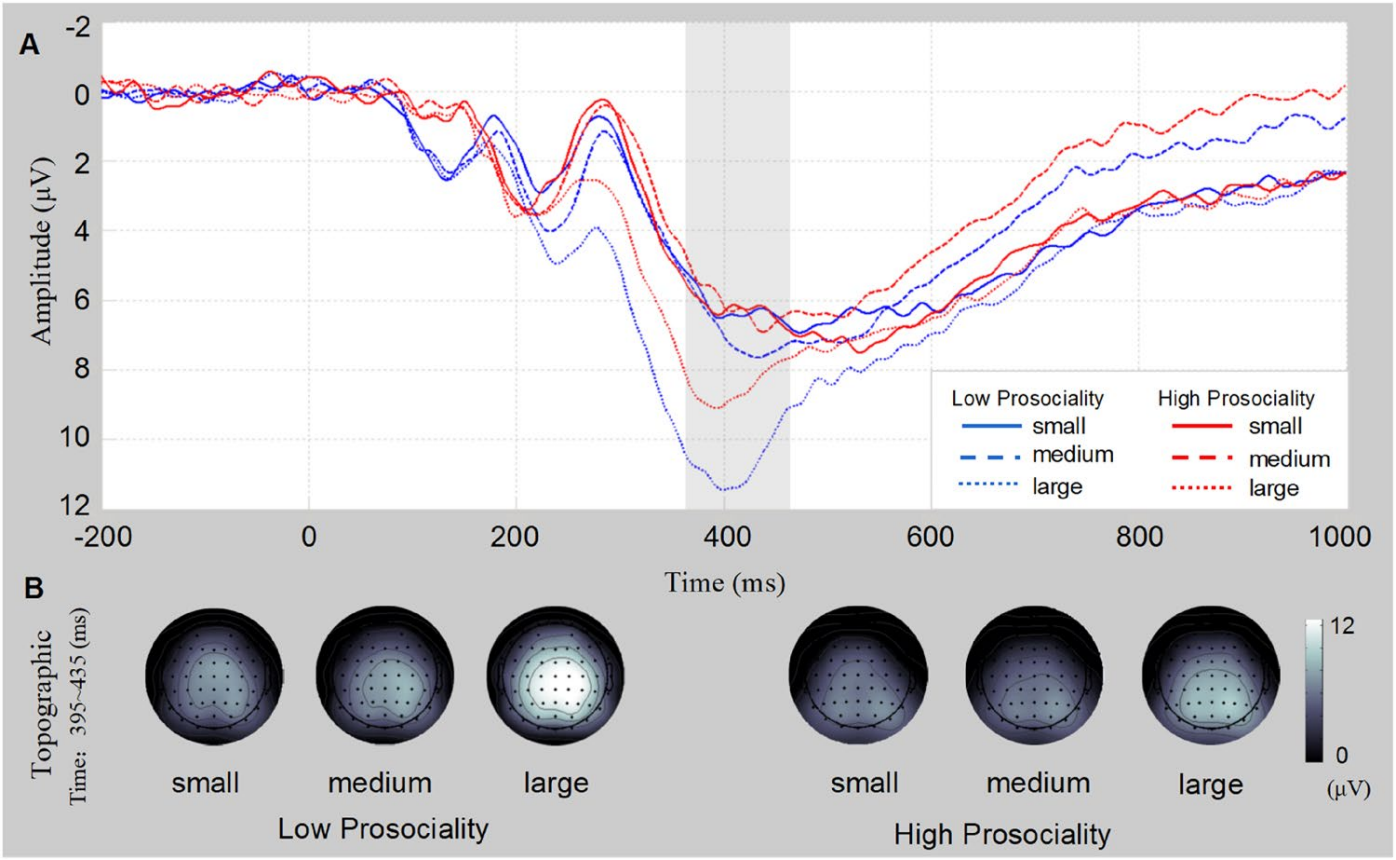
P300 results. (**A**) The mean waves of nine electrodes are shown. The gray area represents the selected time window. Small: the individual choose the card containing 0 star. Medium: the individual choose the card containing 1 star. Large: the individual choose the card containing 2 star. (**B**) The differential topographic map is shown.

For the P300 components (see Fig. 4), the main effect of *size* was significant (F(2, 76) = 62.50, *p* < 0.001, *η*_*p*_^2^ = 0.62), as was the main effect of *group* (F(1, 38) = 0.93, *p* = 0.34. *η*_*p*_^2^ = 0.02), and the interaction between *group* and *size* was significant (F(2, 76) = 6.78, *p* = 0.02, *η*_*p*_^2^ = 0.15). Simple effect analysis found that the amplitude of the P300 in the high prosocial group under the large condition was significantly lower than that of the low prosocial group (*t*(38) = 2.00, *p* = 0.05, Cohen's *d* = 0.65). The difference between the high and low prosocial groups was not significant under the medium condition (*t*(38) = 0.88, *p* = 0.39, Cohen’s *d* = 0.26) or small condition (*t*(38) = 0.24, *p* = 0.81, Cohen’s *d* = 0.08).

In addition, considering the waveforms between 100 and 200 ms for the two groups are different. FRN and P300 components were analyzed by taking the average amplitude of those time period as a covariable, and the results were consistent with the original results (see supplementary material).

### Correlation result

**Figure 5:**
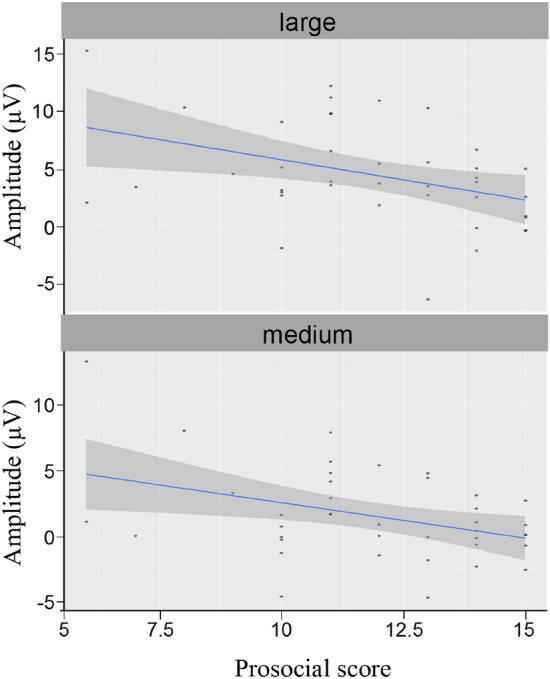
The correlation between prosociality score and FRN amplitude. The horizontal coordinate is the prosocial score, and the total coordinate is the amplitude of FRN.

For the correlation between prosocial score and FRN amplitude (see Fig. 5), there was a significant correlation between prosociality and FRN amplitude in the large (*r* = − 0.40, *p* = 0.012) and medium conditions (*r* = − 0.39, *p* = 0.014), but not in the small condition (*r* = − 0.29, *p* = 0.10).

## Discussion

In this study, we investigated the effect of prosociality alone on outcome evaluation without social comparison or reward processing in face-to-face competition tasks. The results showed that prosociality and size had interactive effects on FRN and P300 components. In other words, in the face-to-face competition task, the amplitude of FRN induced by high-prosocial individuals was significantly negative than that of low-prosocial individuals in the face of medium and large outcome feedback, However, there was no significant difference in FRN between high- and low-prosocial individuals in the face of small outcome feedback. The amplitude of P300 induced in high-prosocial individuals was smaller than that of low-prosocial individuals in the face of large outcome feedback. However, there was no significant difference in P300 between high- and low-prosocial individuals in the face of small and medium outcome feedback. In addition, prosocial scores were significantly negatively correlated with FRN amplitudes when facing medium and large outcome feedback.

Reinforcement learning theory of FRN^14^ states that unexpected results induce more negative FRN than expected results, indicating that FRN is sensitive to expected errors. Oliveira et al.^41^ found that if an individual’s expected result is negative, FRN will be induced, even if the actual result is positive. In other words, regardless of whether the actual result is good or bad, as long as the expected result is different from the actual result, the FRN effect occurs. This indicates that, in this study, the expectation of high-prosocial individuals for the size of the outcome is relatively small compared with that of low-prosocial individuals; that is, high-prosocial individuals expect a small outcome, which leads to a more negative FRN amplitude of prosocial individuals when the result feedback is a larger outcome. This is consistent with the concept of prosociability, in which a person allow others to benefit at their own expense^11,12^. In addition, recent studies suggest that FRN representation outcome reward prediction error is signed^42^. Specifically, FRN is not only composed of negative component, but also contains a positive component. When faced with good outcome (reward), the FRN is driven by positive components^43^. This suggests that in the face of large outcome feedback in current study, low prosocial individuals may be more satisfied with the outcome, leading to a more positive FRN component.

The emotional motivation theory of FRN also provides support for current research, which believes that FRN components reflect people's subjective attitude towards objective results and are influenced by motivational factors^18^. For example, Lei et al.^3^ found that under the condition of low effort, the loss of reward-induced FRN with peer participation was significantly smaller than that without peer participation. The authors explain that individuals sacrifice their own interests to maintain good relationships.In the current study, individuals had to compete with their peers for prizes. When an individual receives a larger number of stars, it indicates that he or she will have a higher probability of outperforming his or her partner, leading to a situation where he or she gets the reward and others lose the reward. This is undesirable for highly prosocial individuals, as it results in a more negative FRN amplitude. Similar phenomena have been reported previously.

This selflessness is not endless, but is limited. In the current study, the main effect of size still existed; that is, both in high- and low-prosocial individuals, the FRN amplitude induced by small outcome was more negative than that induced by large outcome. This indicated that high-prosocial individuals also expected a larger number of stars, which indicates that high-prosocial individuals also expect high scores, but their expectations are lower than those of low-prosocial individuals. At the same time, the FRN amplitude induced by small outcome showed no difference between the high- and low-prosocial groups. Similarly, Lei et al.^3^ found that when individuals put in high effort, the FRNs of loss did not differ significantly between peer participation and without peer participation.

In the current study, the results for P300 were similar to those for FRN components. Researchers generally agree that P300 is associated with the allocation of attention^2,40^ and a high level of emotional evaluation^23,43^; for example, attractive faces induce a larger P300 than unattractive faces^22,44^. Reward brain regions, such as the ventral striatum, are activated by receiving more rewards than others^45^; therefore, in this experiment, having more stars than others may be regarded as a reward with greater motivational significance that captures more attention resources. Studies have found that the P300 is sensitive to the valence and size of the results, and positive feedback induces a larger P300 amplitude than negative feedback^46,47^. Therefore, in the current study, in the face of outcome feedback, larger starts brought stronger positive feedback and higher pleasure to low-prosocial individuals than to high-prosocial individuals.

Contrary to the current research conclusion, in the study of Wang et al.^32^, compared with other individuals, prosocial individuals showed the largest amplitude of FRN when their peers gained rewards while they lost rewards. This may be related to the experimental conditions. In a study by Wang et al.^32^, a social dilemma task was adopted. It is only when a partner betrays that the partner gains the reward. The authors explained that prosocial groups are less tolerant of betrayal than other groups. At the same time, social comparison and reward processing were also involved in their study, which led to the main effect of P300 on valence being only observed in their results. As previous studies have found that the P300 is sensitive to the valence and size of results, positive feedback induces a larger P300 amplitude than negative feedback^2^. In addition, Wang et al.^32^ used the social value orientation scale in their study, in which prosocial refers to common prosperity. This is different from the prosociality of the current study.

Finally, the current study is the first to find that individual prosocial traits can regulate outcome evaluation. This moderating effect was significantly correlated with prosocial traits. This suggests that prosocial traits have a linear effect on FRN components, rather than a certain score. This helps us better understand an individual’s psychological process of outcome evaluation. Prosocial traits should be considered in future outcome evaluation studies. Future studies should examine the role of prosocial traits in different environments, such as tasks with different peers, task (cooperation and competition), and individual emotions.

## Supplementary Information

Supplementary Information.

## Data Availability

The datasets generated- and/or analysed during the current study are not publicly available due to ongoing analysis for a follow-up study, but are available from the corresponding author upon reasonable request.
