# Supplementary material for: Prosociality moderates outcome evaluation in competition tasks
Source: Sci Rep. 2022 Jul 6;12:11397. doi: 10.1038/s41598-022-15570-3 (PMC9259582; doi:10.1038/s41598-022-15570-3)
Supplement: Supplementary file 1 — Supplementary Information. [file 41598_2022_15570_MOESM1_ESM.docx]

For the FRN components, a 3 within-subject (size: small, medium, and large) x 2 between-subject (group: high prosocial, low prosocial) repeated measures ANOVA was performed and the average amplitude of N1 (120 - 140 ms) as a covariable. The results showed that the main effect of *size* was significant (F (2,70) = 12.00, *p* < 0.001, *η_p_*^2^ = 0.33), as was the main effect of *group* (F (1,35) = 10.87, *p* = 0.02. *η_p_*^2^ = 0.28). In addition, the interaction between *group* and *size* was significant (F (2,70) = 6.50, *p* = 0.03, *η_p_*^2^ = 0.16). Simple effect analysis revealed that the amplitude of the high prosocial group under the large condition was significantly more negative that of the low prosocial group (F (1,37) = 11.56, *p* = 0.02. *η_p_*^2^ = 0.24), and the amplitude of the FRN in the high prosocial group under the medium condition was significantly more negative that of the low prosocial group (F (1,37) = 6.45, *p* = 0.02. *η_p_*^2^ = 0.15). However, the difference between high and low prosocial groups was not significant under the small condition (F (1,37) = 1.13, *p* = 0.29. *η_p_*^2^ = 0.03).

For the P300 components, a 3 within-subject (size: small, medium, and large) x 2 between-subject (group: high prosocial, low prosocial) repeated measures ANOVA was performed and the average amplitude of 100 - 200 ms as a covariable.The results showed that the main effect of *size* was significant (F (2,70) = 27.89, *p* < 0.001, *η_p_*^2^ = 0.44), as was the main effect of *group* (F (1,35) = 37.66, *p* < 0.001, *η_p_*^2^ = 0.52). In addition, the interaction between *group* and *size* was significant (F (2,70) = 3.79, *p* = 0.03, *η*^2^ = 0.10). Simple effect analysis revealed that the amplitude of the high prosocial group under the large condition was marginal significantly more negative that of the low prosocial group (F (1,37) = 3.28, *p* = 0.08. *η_p_*^2^ = 0.08), and the amplitude of the high prosocial group under the medium condition was significantly more negative that of the low prosocial group (F (1,37) = 0.45, *p* = 0.512. *η_p_*^2^ = 0.01). However, the difference between high and low prosocial groups was not significant under the small condition (F (1,37) = 0.06, *p* = 0.94. *η_p_*^2^ = 0.003).
